# Cellular stress response mechanisms of Rhizoma coptidis: a systematic review

**DOI:** 10.1186/s13020-018-0184-y

**Published:** 2018-06-07

**Authors:** Jin Wang, Qian Ran, Hai-rong Zeng, Lin Wang, Chang-jiang Hu, Qin-wan Huang

**Affiliations:** College of Pharmacy, Chengdu University of Traditional Chinese Medicine, No. 1166, Liutai Road, Wenjiang District, Chengdu, 611137 China

**Keywords:** Rhizoma coptidis, Alkaloids, Pathways, Diseases

## Abstract

Rhizoma coptidis has been used in China for thousands of years with the functions of heating dampness and purging fire detoxification. But the underlying molecular mechanisms of Rhizoma coptidis are still far from being fully elucidated. Alkaloids, especially berberine, coptisine and palmatine, are responsible for multiple pharmacological effects of Rhizoma coptidis. In this review, we studied on the effects and molecular mechanisms of Rhizoma coptidis on NF-κB/MAPK/PI3K–Akt/AMPK/ERS and oxidative stress pathways. Then we summarized the mechanisms of these alkaloid components of Rhizoma coptidis on cardiovascular and cerebrovascular diseases, diabetes and diabetic complications. Evidence presented in this review implicated that Rhizoma coptidis exerted beneficial effects on various diseases by regulation of NF-κB/MAPK/PI3K–Akt/AMPK/ERS and oxidative stress pathways, which support the clinical application of Rhizoma coptidis and offer references for future researches.

**Electronic supplementary material:**

The online version of this article (10.1186/s13020-018-0184-y) contains supplementary material, which is available to authorized users.

## Background

**Fig. 1:**
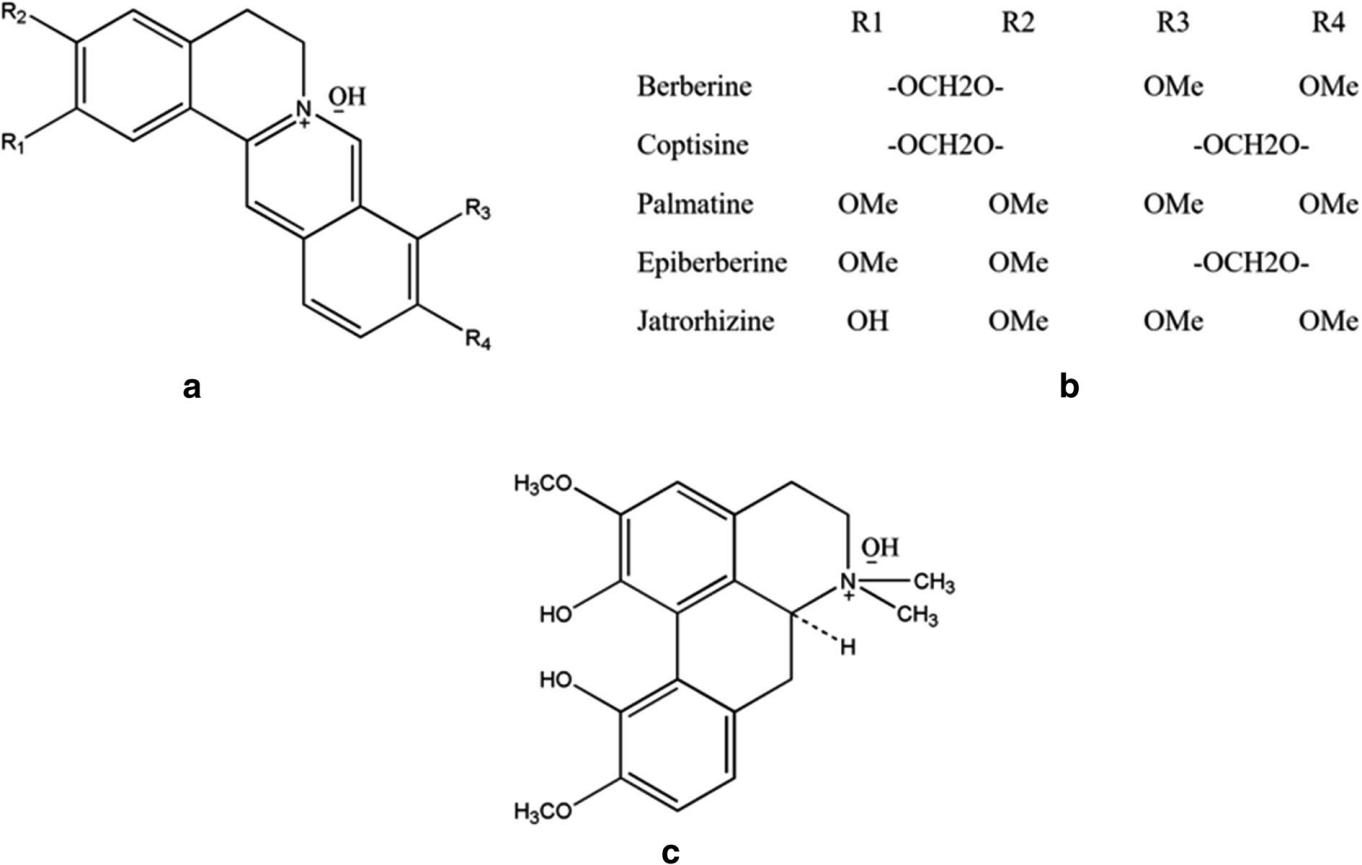
Structures of alkaloids involved in this review. **a** The parent structure of main alkaloids in Rhizoma coptidis. **b** Substituents of the five isoquinoline alkaloids. **c** The molecular structure of magnoflorine

Rhizoma coptidis has been historically well-used as a heat-clearing drug in China. There are many researches on Rhizoma coptidis, but most of them are only a small part of the whole molecular mechanisms. So we need to summarize these researches and find out some rules, which have guiding significance for the further research of Rhizoma coptidis. Modern pharmacological studies have demonstrated that Rhizoma coptidis possesses multiple properties, including neuroprotection [1], anti-inflammation [2–4], antioxygenation [5, 6], anti-cancer [7], anti-atherosclerosis [8], anti-diabetes [9] and anti-obesity [3], etc. These effects of Rhizoma coptidis are attributed to its alkaloid components, especially isoquinoline alkaloids [10]. We chose five isoquinoline alkaloids (berberine, coptisine, palmatine, jateorrhizine, epiberberine) and one aporphine alkaloid (magnoflorine) of Rhizoma coptidis as research goals (Fig. 1).

When the body is under external pressure, cells produce stress responses, defense reactions to resist pressure damage, including NF-κB, MAPK, Akt, AMPK, ERS and oxidative stress signaling pathways [11–14]. NF-κB pathways are associated with immunity, inflammation and cell survival. MAPK pathways refer to various cellular functions, including cell proliferation, differentiation and migration. Akt pathways have effects on apoptosis, protein synthesis, metabolism and cell cycle. AMPK pathways are energy regulation pathways. AMPK pathways inhibit biosynthetic pathways with energy consumption, such as protein, fatty acid and glycogen synthesis. ERS pathways are triggered by the unbalance of ER environment, including hypoxia, disturbance of Ca^2+^ homeostasis and glucose starvation. Oxidative stress pathways are caused by the imbalance of oxidation and antioxidation in body. Oxidative stress pathways regulate redox balance by Nrf2 and other ways.

The theory of traditional Chinese medicine is more from experience and inference, still lack of sufficient scientific basis, and cannot provide a modern scientific basis for overseas popularization and clinical use. Traditional Chinese medicine has multi-components, multi targets. Their complex components have different pharmacokinetic characteristics and complex interactions. The mechanism of Chinese medicine is complex and systematic. As a traditional Chinese medicine, Rhizoma coptidis has excellent therapeutic effects on various diseases, but the underlying systematic molecular mechanisms are still far from being fully elucidated. In this review, we sorted out the relationship of Rhizoma coptidis among components, diseases and NF-κB/MAPK/PI3K/Akt/AMPK/ERS/oxidative stress pathways, systematically studying on how Rhizoma coptidis exerts beneficial effects to various diseases, which supported the clinical application of Rhizoma coptidis and provided references for the future researches.

## The effects of Rhizoma coptidis on NF-κB/MAPK/PI3K–Akt/AMPK/ERS and oxidative stress pathways

### Molecular mechanisms of Rhizoma coptidis inhibiting NF-κB pathways

NF-κB pathways, expressing in all nucleated cells, participate in various diseases by regulation of cellular immunity, proliferation, differentiation and apoptosis, etc. [15]. NF-κB pathways can be mainly activated by two pathways. The canonical pathways are triggered by TNF-α, IL-1β or viral infections. The activation of IKK and the degradation of IκB-α play key roles in the regulation of canonical pathways. TNF-α, IL-1β or LPS activate IKK and lead the degradation of IκB-α and p50/p65 NF-κB dimer entering the nucleus for DNA transcription [16]. In atypical pathways, the activation of NF-κB is shown to be independent from the phosphorylation of IKK and the degradation of IκB-α [17].

**Fig. 2:**
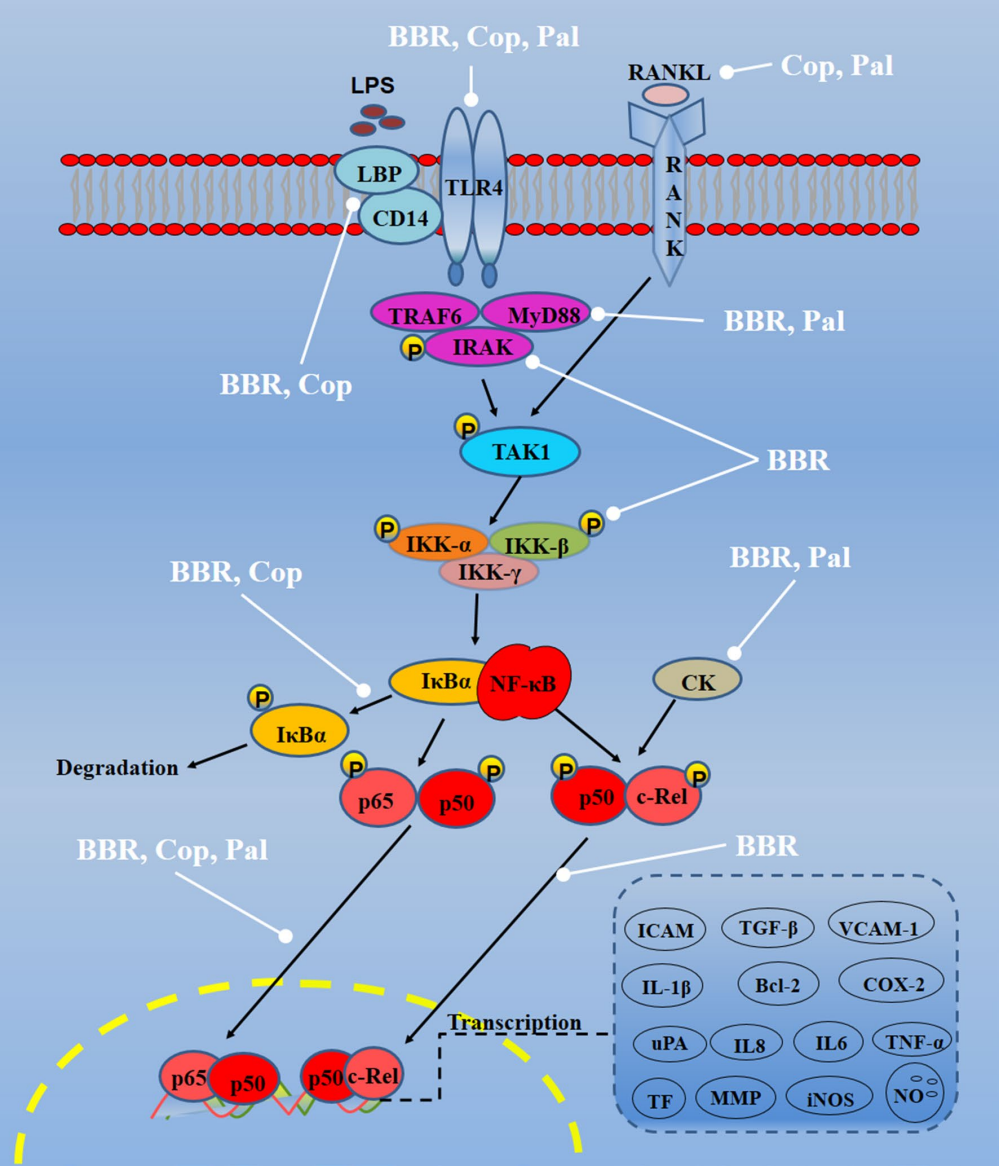
Molecular mechanisms of Rhizoma coptidis on NF-κB pathways (—• indicates inhibition/reduction, arrow indicates promotion/stimulation). NF-κB pathways can be suppressed by Rhizoma coptidis at least four ways, including inhibition of the activity of membrane receptors (i.e., TLR4, CD14), inhibition of the phosphorylation of nuclear factor kappa-B kinase **(**IKK), inhibition of the degradation of NF-κB inhibitor alpha (IκBα), inhibition of activated NF-κB into the nucleus and its binding activity to DNA

Rhizoma coptidis can regulate NF-κB pathways partly as Additional file 1: Table S1 and Fig. 2. Rhizoma coptidis regulates the NF-κB pathways as follows: (A) inhibition of the activity of membrane receptors, including TLR4, CD14; (B) inhibition of the phosphorylation of nuclear factor kappa-B kinase **(**IKK); (C) inhibition of the degradation of NF-κB inhibitor alpha (IκBα); (D) inhibition of activated NF-κB into the nucleus and its binding activity to DNA. Rhizoma coptidis exerts anti-inflammatory and anti-apoptotic effects through the above-mentioned ways, thereby has potential effects to diabetes, osteoarthritis, etc. Berberine reduces the levels of IL-1β, TNF-α in aortic sera and the mRNA expressions of NF-κBp65, iNOS, ICAM-1, IL-6 also reduced by inhibiting the migration of NF-κB to the nucleus in atherosclerotic mice [8, 18]. Coptisine reduces the expression of proinflammatory cytokines including TNF-α, IL-1β and IL-6 in ApoE−/− mice partly by inhibiting NF-κB activation [19].

### Molecular mechanisms of Rhizoma coptidis inhibiting MAPK pathways

MAPK (Mitogen activated protein kinases) pathways play important roles in inflammatory response, cell proliferation, differentiation and apoptosis, etc., mainly through three pathways, ERK (extracellular regulated protein kinases), JNK (c-Jun N-terminal kinase) and p38 MAPK [20, 21]. The activations of MAPK pathways are very conservative. Each MAPK is activated by a specific MAPK kinase. MAPK are the pivots of each pathways. MAPKK kinase (MAP3K or MKKK) and MAPK kinase (MAP2K or MKK) of three pathways have different substrates and intermolecular interconnections, as well as scaffolding proteins [22, 23]. The ERK pathway is activated by mitogen, such as growth factor, platelet-derived growth factor and insulin, and plays important roles in regulating cell growth, survival and differentiation. JNK and p38MAPK pathways weakly activated by mitogen, but can be strongly activated by stress signals, including TNF-α, IL-1β and ultraviolet irradiation, causing inflammatory responses and participating in cell apoptosis [24]. Three pathways can be adjusted specifically. For example, berberine can effectively inhibit the activation of ERK in LPS or IFN-γ induced BV-2 microglia, but it has no impact on the phosphorylation of p38 and JNK [25].

**Fig. 3:**
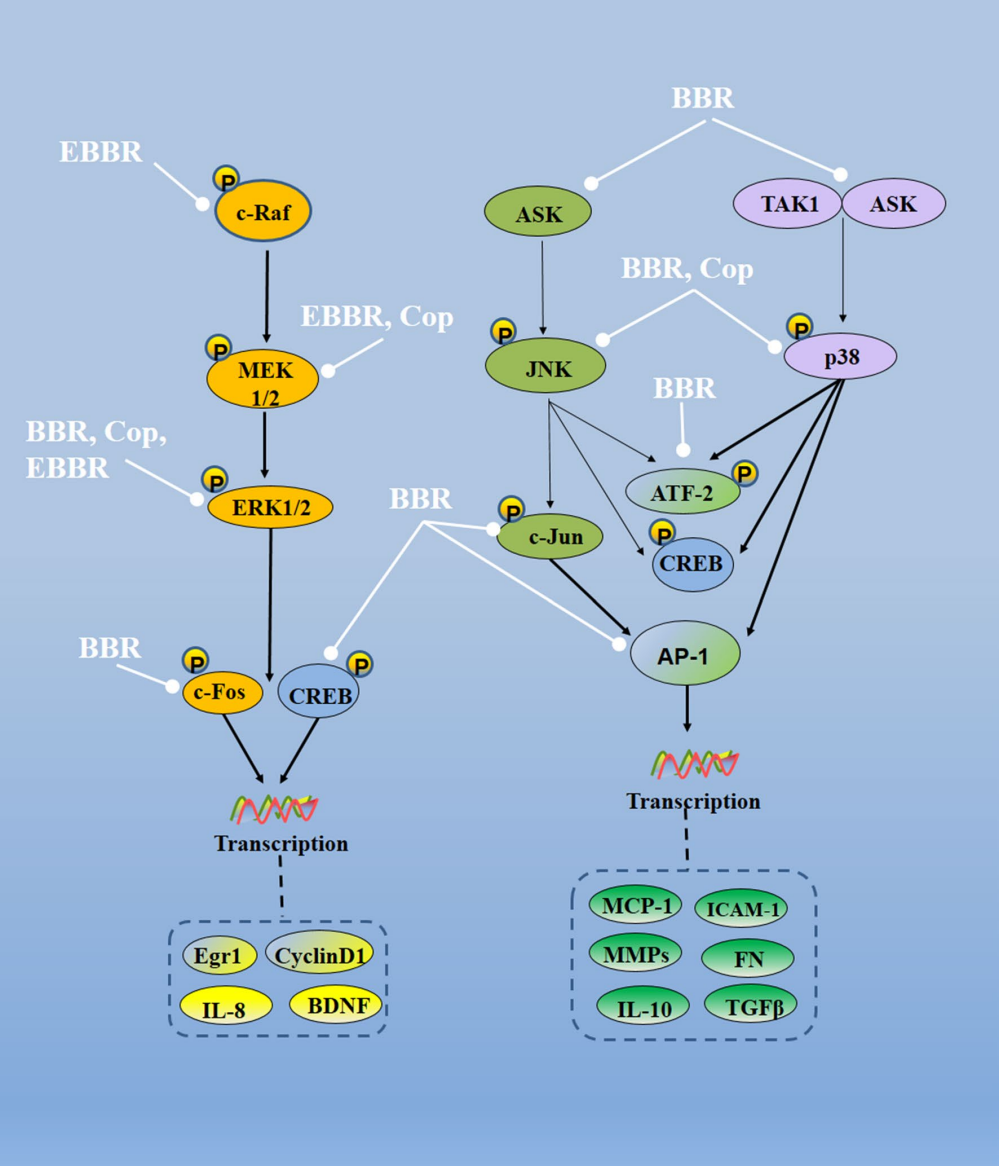
Molecular mechanisms of Rhizoma coptidis on MAPK pathways (—• indicates inhibition/reduction, arrow indicates promotion/stimulation). Rhizoma coptidis can modulate MAPK pathways via several pathways, including ERK, JNK and p38 pathways. Three pathways can be adjusted specifically by Rhizoma coptidis. Through modulation of various MAPK pathways, Rhizoma coptidis suppresses inflammatory responses causing by TNF-α, IL-1β and ultraviolet irradiation and inhibits of apoptosis

Active components of Rhizoma coptidis, especially berberine, can regulate the differentiation, proliferation and apoptosis of cells by inhibition of different MAPK pathways as Additional file 2: Table S2 and Fig. 3, resulting in neuroprotective effect, anti-inflammation, etc. Berberine significantly inhibits the expression of inflammatory cytokines in ARPE-19 cells partly by inhibiting the expressions of p38, ERK1/2, JNK pathways [26]. Berberine can regulate expressions of ERK/P38MAPK/JNK and PI3K–Akt pathways in thyroid cancer cells [27]. Berberine inhibits the activations of MEK/ERK/Egr-1 after mechanical damage of vascular smooth muscle cells in vitro. Once the Egr-1 is enabled, it can regulate the expressions of several genes, including MCP-1, Cyclin D1 and c-Fos [28]. Berberine alleviates vascular inflammation and remodels with metabolic syndrome by inhibiting the activation of p38MAPK, ATF-2 and MMP-2 in the arteries [29]. Berberine has therapeutic effects on diabetic neuropathy through MAPK signaling pathways [30].

### Molecular mechanisms of Rhizoma coptidis regulating AMPK pathways

AMPK pathways exist in most of eukaryotic cells and metabolism related tissues and organs [31]. AMP-activated protein kinase (AMPK) plays important roles in multiple metabolic pathways, including regulating movement, nutrition and hormonal signals at cellular level, so as to control energy consumption and substrate utilization of whole body and maintain the balance of energy metabolism [32, 33]. AMPK promotes glucose uptake and transport by regulating the expression of GLUT4, including promoting GLUT4 translocation and unlocking GLUT4 gene expression [34, 35]. AS160 is a downstream target of AMPK. Phosphorylation of AS160 promotes the translocation of GLUT4 and glucose uptake [36]. PFK (6-phosphofructo-2-kinase) is a speed limiting enzyme of glycolysis. AMPK regulates the phosphorylation of PFK2 [37]. AMPK also can inhibit the activation of FAS, ACC, etc., to inhibit gluconeogenesis and glycogen formation [38, 39]. AMPK participates in the regulation of lipid metabolism, which is related to SREBP1C [40]. AMPK plays a major role in the development of insulin resistance [41]. AMPK can regulate the secretion of insulin and homeostasis of pancreatic β cells [42]. AMPK also plays significant roles in various inflammatory diseases with the function of anti-inflammation [43].

**Fig. 4:**
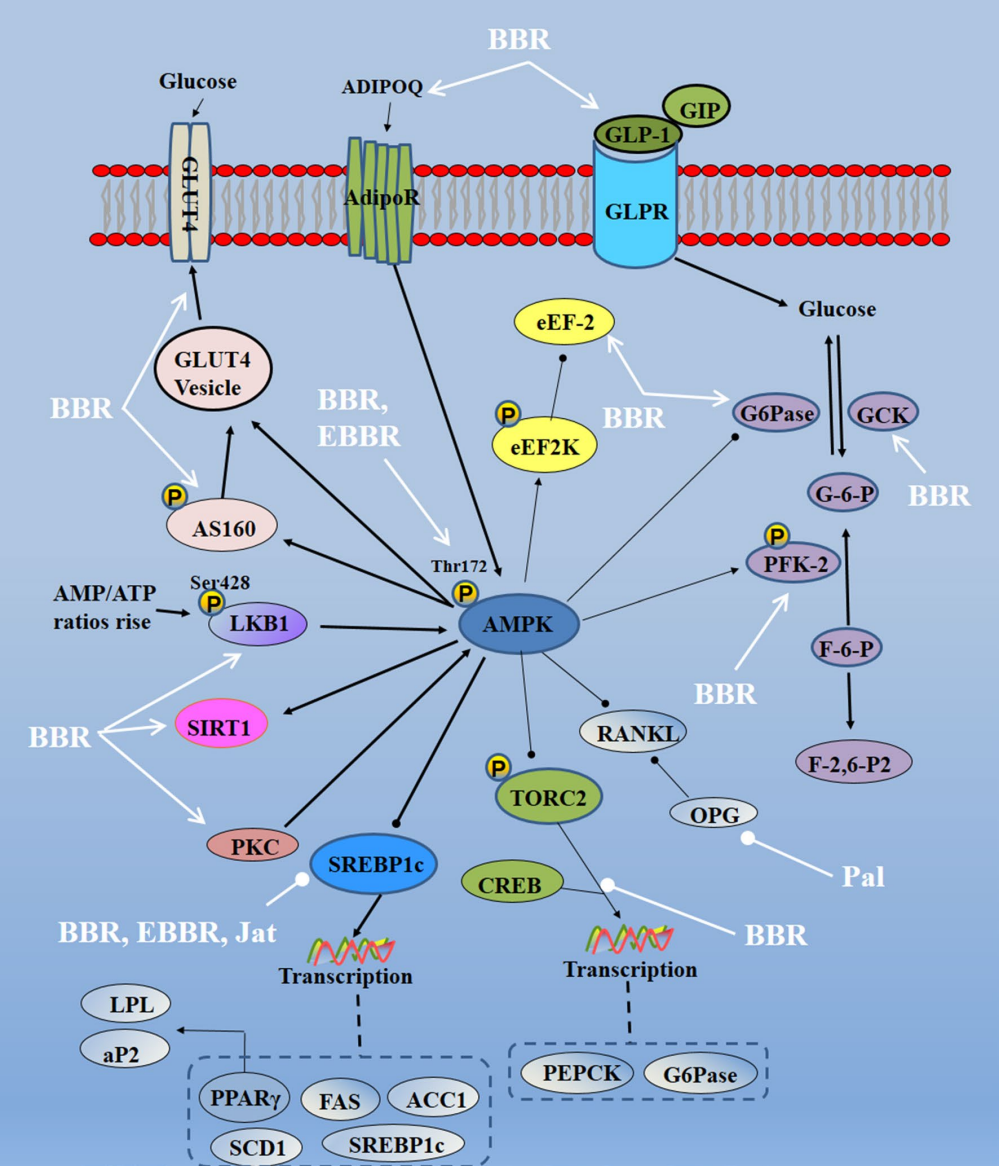
Molecular mechanisms of Rhizoma coptidis on AMPK pathways (—• indicates inhibition/reduction, arrow indicates promotion/stimulation). Rhizoma coptidis can activate AMPK pathways via modulating upstream targets (i.e., LKB1). Once AMPK is activated, downstream signals of AMPK will be stimulated to demonstrate multiple biological effects, including anti-obesity, anti-diabetes, etc.

Rhizoma coptidis can regulate AMPK pathways partly as Additional file 3: Table S3 and Fig. 4. Active components of Rhizoma coptidis activate AMPK partly by activating the upstream targets, including LKB1 [25]. After activating AMPK, Rhizoma coptidis exerts its function as follows: (A) activation of AS160 and GLUT4 to promote glucose transportation; (B) regulation of PFK-2 to promote glycolysis; regulation of FAS and ACC to inhibit gluconeogenesis and glycogen formation; (C) inhibition of SREBP1c and its downstream PPARγ, FAS, and ACC1 to anti-obesity. By those ways, Rhizoma coptidis has therapeutic effects on cerebral ischemia [44], brain injury [45], diabetes [46], and obesity [47], etc.

### Molecular mechanisms of Rhizoma coptidis regulating PI3K–Akt pathways

The full activations of the PI3K–Akt pathways are complex processes. Akt (protein kinase B) can be phosphorylated by PDK1 and PDK2, or directly phosphorylated by phosphatidylinositol 3′-kinase (PI3K) in residues Thr-308 and Ser-473 [48]. Once the Akt is activated, it can regulate the expressions of downstream factors. Akt can inhibit GSK-3β activity by increasing its phosphorylation and there has shown that GSK-3β plays an important role in cellular survival and metabolism [49]. Akt inhibits cell apoptosis by regulating the expressions of BAD (Bcl-2-associated death promoter), Bcl-2, procaspase-9, and caspase-9 [50, 51]. Akt regulates cell growth by controlling the expression of mTORC1 (mTOR complex 1) that controls the initiation of translation and synthesis of ribosome [52]. Akt participates in the process of nutrient absorption and metabolism, such as by regulation of GLUT4 and HIF-1α to mediate insulin stimulation and glucose absorption. Akt is also involved in angiogenesis and cell migration [53–56].

**Fig. 5:**
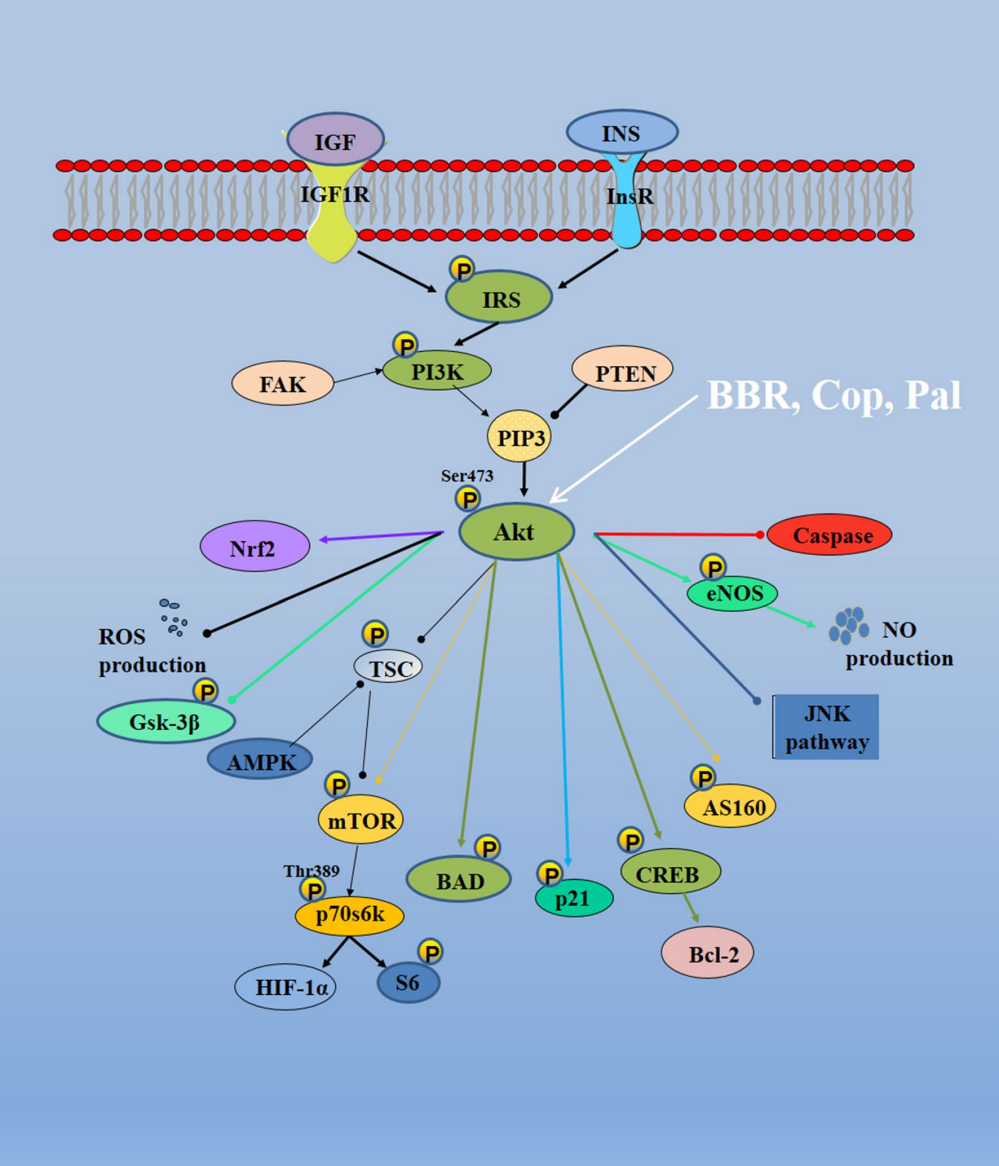
Molecular mechanisms of Rhizoma coptidis on PI3K–Akt pathways (—• indicates inhibition/reduction, arrow indicates promotion/stimulation). By modulating upstream targets (i.e., IRS, PI3K), Rhizoma coptidis increases Akt ser473 phosphorylation. Once Akt is activated, downstream signals (i.e. GSK3β, mTOR, Nrf2) will be stimulated to demonstrate multiple biological effects, including decrease in oxidative stress, inhibition of apoptosis and suppression of inflammation

Rhizoma coptidis can regulate PI3K–Akt pathways partly as Additional file 4: Table S4 and Fig. 5. Berberine can be utilized to treat skin pigmentation and prevent cardiac dysfunction by regulating PI3K/Akt/GSK3 pathway [57, 58]. Berberine regulates cardiac fibroblast proliferation, collagen synthesis, cytokine secretion and induces the apoptosis of gastric cancer cells by Akt/mTOR/p70S6K pathway [59, 60]. Berberine protects endothelial progenitor cells from TNF-α by increasing the expressions of PI3K/Akt/eNOS [61]. PI3K–Akt pathways are widely expressed in the development of central nervous system [62]. BBR regulates the PI3K/Akt/GSK3β pathway at the early stage of neuronal polarization and promotes AMPK activation in low energy state to mediate the growth of neurite and affect the stability of cellular cytoskeleton [32]. Also, BBR induces the activation of PI3K/Akt/Nrf2 to protect dopaminergic SH-SY5Y neuron cells, NSC34 motoneuron cells and astrocytes [1, 63, 64].

### Molecular mechanisms of Rhizoma coptidis regulating ERS pathways

**Fig. 6:**
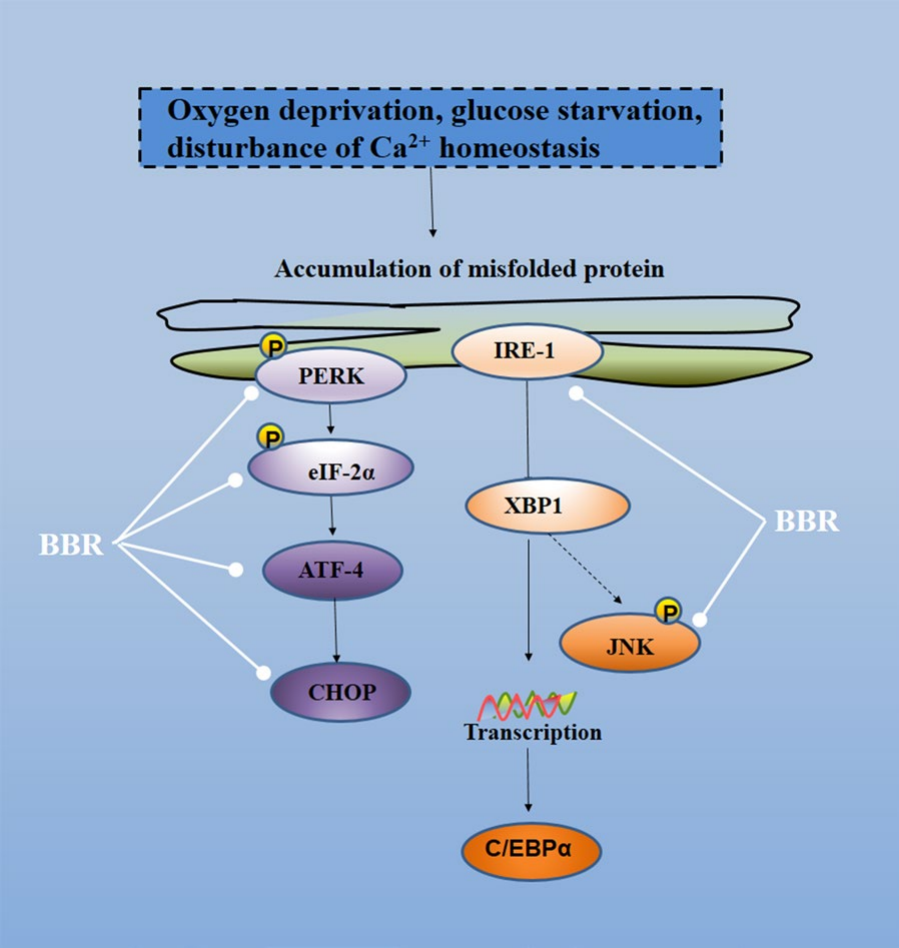
Molecular mechanisms of Rhizoma coptidis on ERS pathways (—• indicates inhibition/reduction, arrow indicates promotion/stimulation). ERS pathways can be inhibited by Rhizoma coptidis at least two ways, including inhibition of the expression of IRE-1 and inhibition of the phosphorylation of PERK

Endoplasmic reticulum (ER) is an important subcellular organelle that completes the protein folding and modification. When cells are subjected to intensive stimuli, such as oxygen deprivation, glucose starvation, disturbance of Ca^2+^ homeostasis, the endoplasmic reticulum will accumulate of unfolded/misfolded protein and induce endoplasmic reticulum stress (ERS). Then transcription factor 6 (ATF6), inositol-requiring protein-1 (IRE1) and PKR-like ER kinase (PERK) will be activated to reduce endoplasmic reticulum burden and maintain endoplasmic reticulum homeostasis [11, 65]. Recent studies have shown that ERS pathways play critical roles in the pathogenesis of obesity, insulin resistance and T2DM [66]. Berberine is localized both in the nucleus and ER [32]. Rhizoma coptidis, through down-regulation of proteins in ERS pathways, including PERK, IRE-1α, eIF2α and CHOP, has therapeutic effects on ER stress-associated diseases, including obesity, inflammation and diabetes (Additional file 5: Table S5; Fig. 6). Berberine protects HepG2 cells from ER stress damage by inhibiting the expressions of PERK, eIF2α in ERS pathways [66]. Berberine exerts neuroprotective effects by down-regulation of ERS related proteins [67]. Also, berberine inhibits inflammatory cytokines induced inflammation in human intestinal epithelial cells through down-regulation of ERS related proteins [68]. In addition, berberine can selectively up-modulation of ERS components, CHOP, ATF3, ATF4 and TRB3 with a concomitant down-modulation of C/EBPα and PPARγ to alleviate ER stress [69].

### Molecular mechanisms of Rhizoma coptidis regulating oxidative stress pathways

Low levels of ROS/RNS are necessary for cell proliferation and differentiation. Excessive levels of ROS/RNS directly destruct of cell membrane, DNA and proteins, resulting in cell function damage, proliferation inhibition and apoptosis [70]. ROS are radical species, including O^2−^, H_2_O, OH•, peroxylradicals, etc. RNS are radical species, including NO and ONOO^−^, etc. [71, 72]. Under normal condition, antioxidant proteins, such as glutathione (GSH), ROS metabolic enzymes, including glutathione peroxidase, catalase and superoxide dismutase (SOD), are in balance. When this redox homeostasis is unbalanced, oxidative stress will be activated and participate in the occurrence and development of various diseases [72].

Oxidative stress interacts with other pathways [65]. NF-κB, MAPKs, AP-1, HIF, PI3K/Akt are redox-sensitive transcription factors. ROS activates NF-κB pathways through activation of IκB kinase, regulation of the chromatin remodeling, coactivator recruitment and DNA binding ability of NF-κB [72, 73]. Berberine down regulates ROS-related ASK1, p38/JNK, and NF-κB pathways in osteoarthritis synovial fibroblasts [74]. Berberine also prevents ROS dependent and JNK driven apoptosis in bone marrow mesenchymal stem cells [70].

Nrf2 is an important transcription factor that regulates the balance of redox in cells. Under normal physiological conditions, Nrf2 binds to Keap1 in cytoplasm. After activation, Nrf2 separates from Keap1 and binding to ARE site to activate the downstream promoters and proteins, including NQO1, HO-1, GST, thioredoxin [75, 76]. In SH-SY5Y cells, Nrf2 siRNA abolished BBR-induced HO-1, neurite outgrowth and ROS decrease, which indicates that berberine is a Nrf2 activator against glucose neurotoxicityas [49]. Also, berberine, a PI3K activator, can regulate the oxidative stress by regulating the PI3K–Akt–Nrf2 pathway. PI3K–Akt and MAPKs pathways are important for BBR to induce Nrf2 translocation and HO-1 expression [21, 49, 64]. Coptisine also can activate Nrf2 and its upstream targets, including Akt and JNK, thus to regulate expressions of NQO1, ROS, GSH SOD and GPx in HepG2 cells [77].

Therefore, Rhizoma coptidis can regulate oxidative stress partly as Additional file 6: Table S6. Rhizoma coptidis can regulate oxidative stress by following: (1) regulation of ROS/RNS radical species, including H_2_O_2_, ONOO^−^; (2) regulation the productions of antioxidant proteins, including ROS metabolic enzymes; (3) regulation the expressions of redox-sensitive transcription factors, including NF-κB, MAPKs, AP-1, HIF, PI3K/Akt; (4) stimulating the translocation activity and nuclear accumulation of Nrf2, as well as promoting the Nrf2-DNA binding activity.

## NF-κB/MAPK/AMPK/PI3K–Akt/ERS/oxidative stress pathways in disease states

### Cardio-cerebral-vascular system

Rhizoma coptidis has significant effects on the main pathogenic factors of cardiovascular diseases, including anti-atherosclerosis, lipid lowering and anti-ischemia reperfusion injury. These properties have been attributed to alkaloid components of Rhizoma coptidis, including berberine, coptisine, palmatine, epiberberine, jatrorrhizine and magnoflorine [78, 79].

#### Ischemic–reperfusion injury

Rhizoma coptidis has therapeutic effects on ischemia–reperfusion injury by regulation of Akt/AMPK/p38/ERS/oxidative stress pathways as follows: (1) regulation of Ak/GSK3β/CREB as well as JNK/ERK1/2 to reduce ischemic brain injury [48]; (2) activation of PI3K–Akt, enhancement the accumulation of HIF-1α and promoting of HIF-1α mediated Sphk2 transcriptional to protect endogenous nerves [44]; (3) activation of AMPK, phosphorylation of Akt, thus exerting protective effects on the non-ischemic regions of the diabetic heart [80]; (4) activation of AMPK and PI3K–Akt–eNOS pathways to reduce myocardial apoptosis induced by ischemia/reperfusion in diabetic rats [81]; (5) regulation of lactate dehydrogenase (LDH), creatine phosphokinase (CK), superoxide dismutase and catalase (CAT) and other antioxidant enzyme activities [82]; (6) P38MAPK is crucial in myocardial ischemia and reperfusion [83]. Rhizoma coptidis also inhibits ischemia–reperfusion injury by blocking the activity of p38MAPK.

#### Obesity

Berberine, epiberberine, coptisine, palmatine, and magnoflorine can inhibit the lipid accumulation significantly in a dose-dependent manner in 3T3-L1 cells. They can down regulate the expression of adipocyte marker proteins, including C/EBP-α and PPAR-γ. The inhibition ability is as follows: coptisine > berberine > epiberberine > palmatine > magnoflorine [84].

AMPK and SREBP-1c are the main therapeutic targets for the treatment of metabolic diseases. AMPK is a kinase responsible for the phosphorylation of SREBP-1c in adipocytes. SREBP-1c is a transcription factor associated with the promoter region of a number of genes related to fat formation. SREBP-1c plays an important role in increasing triglyceride synthesis by increasing numbers of adipose producing genes related to FAS, also by promoting the expression of PPAR-γ [85]. Berberine can induce SREBP-1c phosphorylation by AMPK/Akt phosphorylation, thus inhibiting protein hydrolysate, nuclear translocation and DNA binding ability of SREBP-1c and reduce the expressions of lipogenic genes including FAS, LPL, PPAR-γ, C/EBP-α and ACC1 in a dose-dependent manner [85]. Epiberberine can inhibit lipid accumulation by regulating the expressions of AMPK/Akt and cellular differentiation mediated Raf/MEK1/ERK1/2, and then down regulating the main transcription factors of adipose formation, such as PPAR-c, C/EBP-α, SREBP-1c, and FAS in 3T3-L1 adipocytes [47]. Jatrorrhizine can also down regulate the expressions of SREBP-1c and FAS in the liver of high-fat diet-induced obesity mouse model [86].

### Diabetes and its complications

Berberine, epiberberine, magnoflorine, palmatine, jatrorrhizine and coptisine have antidiabetic potential [87, 88]. Jatrorrhizine, palmatine and magnoflorine have potential therapeutic effects on diabetic complications such as retinopathy, cataract, neuropathy, and kidney disease [89].

#### Insulin resistance and glucose metabolism disorder

Rhizoma coptidis plays a therapeutic role in insulin resistance partly by regulating the TLR4/JNK/NF-κB pathways and Akt/AMPK/GLP-1/ERS pathways. In the insulin resistance models, Rhizoma coptidis acts on the TLR4/JNK/NF-κB inflammatory pathways and inhibits the activities of MCP-1, IL-6, TNF-α, JNK and NF-κB [9, 90–93]. AMPK also plays an important role in Rhizoma coptidis’s treatments on diabetes [94]. Rhizoma coptidis up-regulates the expression of LKB1, AMPK and inhibits the translocation of TOCR2 into the nucleus in the liver of diabetic rats [46]. Berberine regulates the expression of GLUT4 in insulin-resistant cells with AMPK dependent and Akt independent ways. Akt is an important kinase that mediates glucose metabolism stimulated by insulin, and the deficiency of Akt can lead to glucose metabolism disorder. Rhizoma coptidis acts on Akt pathways by modifying IRS, phosphorylation of downstream Akt and activating PKC to improve insulin signaling cascade [66, 95, 96]. Protein kinase C (PKC) is expressed by the insulin receptor. Berberine can decrease insulin resistance by activating PKC, and inhibitor of PKC can eliminates InsR activation and InsR mRNA transcription induced by berberine [97]. IRS-1 is the principal link between inflammation and insulin resistance [95]. Berberine can improve the IRS-1 level in the brain and restore the expressions of GLUT1 and GLUT3 in the treatment of diabetic animals [98]. Berberine also improves insulin resistance in nonalcoholic fatty liver disease by regulating the expression levels of IRS-2 [95]. Akt induces GLUT translocation to the plasma membrane and regulates downstream targets involved in glycogen synthesis including GSK-3β and glucokinase [99]. Berberine activates Akt and GCK in liver and adipose tissue. The activity of GSK-3β in the liver was also inhibited by berberine [90, 96, 99]. Recent studies have shown that ERS play important roles in obesity, insulin resistance and T2DM. Rhizoma coptidis treats on insulin resistance partly by acting on the ERS pathways, including JNK, PERK, eIF2α and ORP150 to protect cells from ER stress injury [66].

Rhizoma coptidis also treats insulin resistance by activation of GLP-1. Berberine may regulate the secretion of GLP-1 by regulating AMPK [100]. GLP-1 plays an important role in stimulating glucose dependent insulin secretion, inhibiting glucagon release, promoting β cell proliferation, and gastric emptying and food intake [100]. Rhizoma coptidis can increase the expression of GLP in type 2 diabetic rats to stimulate glucose consumption and lower the levels of blood glucose [101]. Berberine also promotes the secretion of GLP-1 by activating TGR5 and bitterer receptor subtype TAS2R38. TGR5 plays an important role in regulating glycolipid metabolism, inhibiting inflammation, and improving kidney disease [101, 102].

Glucose transportation is the speed limiting step of glucose metabolism and can be activated in the peripheral tissue by two different ways: (1) IRS-1/PI3K signal transduction stimulated by insulin; (2) activation of muscle contraction through AMPK [103]. Berberine can improve glucose metabolism by insulin independent way in insulin sensitive cells including HepG2, C2C12, L6, 3T3-L1 cells [91, 104]. Berberine can increase the ratio of AMP/ATP and activate AMPK and Akt pathways to promote acute insulin mediated glucose transportation [101, 103].

#### Diabetic nephropathy

Diabetic nephropathy (DN) is one of the microvascular complications of diabetes and is the main cause of end-stage diabetes [105]. Oxidative stress, ERS and inflammation play important roles in the treatment of DN with Rhizoma coptidis. The levels of AGEs, P-PKC-β and TGF-β increased in injured kidneys. Berberine significantly reduced these levels, but the specific mechanisms remain to be further studied [106].

Rhizoma coptidis can treat diabetic nephropathy by AMPK/NF-κB/MAPK/Akt/oxidative stress/ERS pathways in diabetic nephropathy. (A) Under high glucose conditions in glomerular mesangial cells, berberine activates TGR5 and inhibits NF-κB/AP-1/MAPK pathways, decreasing the expressions of fibronectin (FN), ICAM-1, TGF-β1 and the phosphorylation levels of c-Jun/c-Fos [101, 107, 108]. High glucose can induce the over expressions of TGF-β1 in diabetic animals and patients, leading to renal fibrosis. However, berberine can reduce the expression of TGF-β1 by NF-κB pathways [108]. At the same time, berberine suppresses the p38MAPK pathways to inhibit the formation of fibronectin and collagen, which participate in renal fibrosis [105]. (B) Under high glucose conditions in renal tubular epithelial cells, berberine activates the PI3K/Akt pathways, reducing the expressions of apoptosis related proteins including Bax, cytochrome c, caspase3 and caspase9. Berberine also has effects on the oxidative stress pathways by reducing ROS production, promoting the expressions of GSH, SOD, Nrf2 and OH-1 to protect the renal tubular epithelial cells from apoptosis [75]. In dorsal root ganglion neurons, Berberine can reduce ROS production and rescue mitochondrial dysfunction induced by high glucose by increasing the expression of p-AMPK and Nrf2 [109]. (C) The sertoli cells arrange at the height of the glomerular basement membrane, and the lack of sertoli cells in regeneration is the main restriction of glomerular healing. Berberine can prevent Aldo induced sertoli cells injury and apoptosis by inhibiting the expressions of ERS related protein GRP78 and CHOP [110].

## Discussion and conclusion

As a traditional Chinese medicine, Rhizoma coptidis is widely cultivated in China. It can be employed in infectious diseases, cardiovascular diseases, diabetes and so on. Its new efficacy is also constantly being discovered. In this paper, we summarized its mechanisms from the relationships of effective components, diseases and pathways. We found that various pharmacological effects of Rhizoma coptidis were due to its overall effects on multiple targets and pathways. The single component of the Rhizoma coptidis can act on several pathways at the same time. Multiple effective components of Rhizoma coptidis also play synergistic roles in pathways. In Osteoarthritis articular chondrocytes, berberine activates Akt/p70S6K/S6 pathway to promote protein synthesis, cell survival and matrix production, and also strongly induces differentiation through PI3K/Akt and p38 kinase pathway [111, 112]. Berberine also regulates the formation of osteoclasts through the negative feedback of AK1 and the activation of AMPK [113]. Coptisine inhibits NF-kB activation and the expression of COX-2, MMP-3, MMP-13 in Osteoarthritis articular chondrocytes [114]. Palmatine may attenuate osteoclast differentiation by inhibiting the expressions of OPG, RANK and RANKL [115, 116].

The related signaling pathways of Rhizoma coptidis are complex. It has been studied from the point of network pharmacology and revealed some mechanisms from the body system. But the activations of the corresponding biomarkers are caused by which components, which components regulate the networks or targets and whether there are synergistic/antagonistic effects between these components remain to be further studied. How to integrate the components, pharmacological actions and targets of Rhizoma coptidis is the focus and difficulty of future researches.

Therefore, in order to make full use of Rhizoma coptidis, we should use the modern technical means, such as the combination of the composition-activity relationship with metabolomics, to find the material basis and system mechanisms of Rhizoma coptidis. It will provide important bases for clinical application and comprehensive utilization of Rhizoma coptidis.

## Additional files

**Additional file 1: Table S1.** The effects of Rhizoma coptidis on NF-κB pathways (↓indicates inhibition/reduction, ↑indicates increase).

**Additional file 2: Table S2.** The effects of Rhizoma coptidis on MAPK pathways (↓indicates inhibition/reduction, ↑indicates increase).

**Additional file 3: Table S3.** The effects of Rhizoma coptidis on AMPK pathways (↓indicates inhibition/reduction, ↑indicates increase).

**Additional file 4: Table S4.** The effects of Rhizoma coptidis on PI3K-Akt pathways (↓indicates inhibition/reduction, ↑indicates increase).

**Additional file 5: Table S5.** The effects of Rhizoma coptidis on ERS pathways (↓indicates inhibition/reduction, ↑indicates increase).

**Additional file 6: Table S6.** The effects of Rhizoma coptidis on Oxidative Stress pathways (↓indicates inhibition/reduction, ↑indicates increase).

## Abbreviations

BBR: berberine
Cop: coptisine
Pal: palmatine
EBBR: epiberberine
Jat: jateorrhizine
Mag: magnoflorine
PI3K: phosphatidylinositol 3′-kinase
MyD88: myeloid differentiation primary response protein
IKK: inhibitor of nuclear factor kappa-B kinase
IκBα: NF-κB inhibitor alpha
TLR4: toll like receptor 4
LBP: lipopolysaccharide binding protein
CK: casein kinase
IRAK: interleukin 1 receptor associated kinase
ICAM-1: intercellular adhesion molecule-1
Bcl-2: B-cell lymphoma-2
VCAM-1: vascular cell adhesion molecule-1
u-PA: urokinase plasminogen activator
MMP: matrix metalloproteinase
RANKL: receptor activator for nuclear factor-κ B ligand
mTOR: mammalian target of rapamycin
MEK1/2: mitogen-activated protein kinase kinase 1/2
ASK1: apoptosis signal-regulating kinase 1
Egr1: early growth response protein-1
TAK1: TGFβ-activated kinase 1
MCP-1: monocyte chemoattractant protein-1
CREB: cAMP-response element binding protein
BDNF: brain-derived neurotrophic factor
FN: fibronectin
AP-1: activator protein-1
TGF-β1: transforming growth factor beta 1
ICAM-1: intercellular cell adhesion molecule-1
Cytc: cytochrome c
MMPs: matrix metalloproteinases
ATF-2: activating transcription factor-2
GLUT: glucose transporter
TORC2: CREB-regulated transcription co-activator 2
GCK: glucokinase
ADIPOQ: adiponectin
ACC: acetyl-CoA carboxylase
GLP-1: glucagon-like peptide-1
GIP: gastric inhibitory polypeptide
LKB1: liver kinase B1
PEPCK: phosphoenolpyruvate carboxykinase
G6Pase: glucose-6-phosphatase
eEF2: eukaryotic translation elongation factor 2
SREBP-1c: sterol regulatory element-binding transcription factor-1c
PPARγ: peroxisome proliferator-activated receptor γ
aP2: activating protein 2
LPL: lipoprotein lipase
FAS: fatty acid synthase
OPG: osteoprotegerin
SIRT1: sirtuin 1
IRS: insulin receptor substrate
InsR: insulin receptor
IGF-1: insulin-like growth factor-1
HIF-1α: hypoxia inducible factor-1α
ROS: reactive oxygen species
PTEN: phosphatidylinositol-3,4,5-trisphosphate 3-phosphatase and dual-specificity protein phosphatase
FAK: focal adhesion kinase
eIF2α: eukaryotic initiation factor 2α
IRE-1α: inositol-requiring enzyme-1α
CHOP: C/EBP homologous protein
C/EBPα: CCAAT/enhancer binding protein alpha
GRP78: glucose-regulated protein 78
ATF4: AMP-dependent transcription factor 4
Nrf2: nuclear factor-erythroid 2 related factor 2
HO-1: heme oxygenase-1
GSH: glutathione
SOD: superoxide dismutase
8-OHdG: 8-hydroxy-2 deoxyguanosine
NQO1: NAD(P)H quinone dehydrogenase 1
MPO: myeloperoxidase
ONOO−: peroxynitrite anion
NOX: nicotinamide adenine dinucleotide phosphate-oxidase
GPx: glutathione peroxidase
STZ: streptozotocin

## References

[1] Hsu YY (2012). Berberine activates Nrf2 nuclear translocation and protects against oxidative damage via a phosphatidylinositol 3-kinase/Akt-dependent mechanism in NSC34 motor neuron-like cells. Eur J Pharm Sci.

[2] Yan B (2017). Palmatine inhibits TRIF-dependent NF-kappaB pathway against inflammation induced by LPS in goat endometrial epithelial cells. Int Immunopharmacol.

[3] Zou ZY (2015). Coptisine attenuates obesity-related inflammation through LPS/TLR-4-mediated signaling pathway in Syrian golden hamsters. Fitoterapia.

[4] Gao MY (2014). Berberine inhibits LPS-induced TF procoagulant activity and expression through NF-kappaB/p65, Akt and MAPK pathway in THP-1 cells. Pharmacol Rep.

[5] Zhao H (2013). Berberine suppresses gero-conversion from cell cycle arrest to senescence. Aging.

[6] Yokozawa T (2005). Protective role of Coptidis Rhizoma alkaloids against peroxynitrite-induced damage to renal tubular epithelial cells. J Pharm Pharmacol.

[7] Zhang Y, Liang Y, He C (2017). Anticancer activities and mechanisms of heat-clearing and detoxicating traditional Chinese herbal medicine. Chin Med.

[8] Feng M (2017). Comparative effect of berberine and its derivative 8-cetylberberine on attenuating atherosclerosis in ApoE(−/−) mice. Int Immunopharmacol.

[9] Zhang DS (2015). Effect of berberine on the insulin resistance and TLR4/IKKbeta/NF-kappaB signaling pathways in skeletal muscle of obese rats with insulin resistance. J Sichuan Univ Med Sci Ed.

[10] Meng FC (2018). Coptidis rhizoma and its main bioactive components: recent advances in chemical investigation, quality evaluation and pharmacological activity. Chin Med.

[11] Kültz D (2005). Molecular and evolutionary basis of the cellular stress response. Annu Rev Physiol.

[12] Hardie DG, Ross FA, Hawley SA (2012). AMPK: a nutrient and energy sensor that maintains energy homeostasis. Nat Rev Mol Cell Biol.

[13] Simmons SO, Fan CY, Ramabhadran R (2009). Cellular stress response pathway system as a sentinel ensemble in toxicological screening. Toxicol Sci.

[14] Qi H, Li L, Ma H (2017). Cellular stress response mechanisms as therapeutic targets of ginsenosides. Med Res Rev.

[15] Oeckinghaus A, Hayden MS, Ghosh S (2011). Crosstalk in NF-κB signaling pathways. Nat Immunol.

[16] Gilmore TD (2006). Introduction to NF-kappaB: players, pathways, perspectives. Oncogene.

[17] Esparza-López J (2013). Doxorubicin induces atypical NF-κB activation through c-Abl kinase activity in breast cancer cells. J Cancer Res Clin Oncol.

[18] Wan X (2013). Berberine ameliorates chronic kidney injury caused by atherosclerotic renovascular disease through the suppression of NFkappaB signaling pathway in rats. PLoS ONE.

[19] Feng M (2017). The protective effect of coptisine on experimental atherosclerosis ApoE−/− mice is mediated by MAPK/NF-κB-dependent pathway. Biomed Pharmacother.

[20] Nebreda AR, Porras A (2000). p38 MAP kinases: beyond the stress response. Trends Biochem Sci.

[21] Sun Z, Huang Z, Zhang DD (2009). Phosphorylation of Nrf2 at multiple sites by MAP kinases has a limited contribution in modulating the Nrf2-dependent antioxidant response. PLoS ONE.

[22] Morrison DK, Davis RJ (2003). Regulation of MAP kinase signaling modules by scaffold proteins in mammals. Annu Rev Cell Dev Biol.

[23] Enslen H, Davis RJ (2001). Regulation of MAP kinases by docking domains. Biol Cell.

[24] Kyriakis JM, Avruch J (2001). Mammalian mitogen-activated protein kinase signal transduction pathways activated by stress and inflammation. Physiol Rev.

[25] Lu DY (2010). Berberine suppresses neuroinflammatory responses through AMP-activated protein kinase activation in BV-2 microglia. J Cell Biochem.

[26] Wang Q (2012). Effect of berberine on proinflammatory cytokine production by ARPE-19 cells following stimulation with tumor necrosis factor-alpha. Invest Ophthalmol Vis Sci.

[27] Li L (2017). Berberine could inhibit thyroid carcinoma cells by inducing mitochondrial apoptosis, G0/G1 cell cycle arrest and suppressing migration via PI3K-AKT and MAPK signaling pathways. Biomed Pharmacother.

[28] Liang KW (2006). Berberine suppresses MEK/ERK-dependent Egr-1 signaling pathway and inhibits vascular smooth muscle cell regrowth after in vitro mechanical injury. Biochem Pharmacol.

[29] Li XX (2015). Berberine attenuates vascular remodeling and inflammation in a rat model of metabolic syndrome. Biol Pharm Bull.

[30] Zhou J (2016). Neuroprotective effect of berberine is mediated by MAPK signaling pathway in experimental diabetic neuropathy in rats. Eur J Pharmacol.

[31] Carling D (2011). AMP-activated protein kinase: nature’s energy sensor. Nat Chem Biol.

[32] Lu J (2015). Berberine regulates neurite outgrowth through AMPK-dependent pathways by lowering energy status. Exp Cell Res.

[33] Steinberg GR, Kemp BE (2009). AMPK in health and disease. Physiol Rev.

[34] Lee JO (2012). Metformin regulates glucose transporter 4 (GLUT4) translocation through AMP-activated protein kinase (AMPK)-mediated Cbl/CAP signaling in 3T3-L1 preadipocyte cells. J Biol Chem.

[35] Miyamoto L (2007). Effect of acute activation of 5′-AMP-activated protein kinase on glycogen regulation in isolated rat skeletal muscle. J Appl Physiol.

[36] Schweitzer GG, Arias EB, Cartee GD (2012). Sustained postexercise increases in AS160 Thr642 and Ser588 phosphorylation in skeletal muscle without sustained increases in kinase phosphorylation. J Appl Physiol.

[37] Arden C (2008). A role for PFK-2/FBPase-2, as distinct from fructose 2,6-bisphosphate, in regulation of insulin secretion in pancreatic beta-cells. Biochem J.

[38] Cantó C, Auwerx J (2010). AMP-activated protein kinase and its downstream transcriptional pathways. Cell Mol Life Sci CMLS.

[39] Chen MH, Lin CH, Shih CC (2014). Antidiabetic and antihyperlipidemic effects of *Clitocybe nuda* on glucose transporter 4 and AMP-activated protein kinase phosphorylation in high-fat-fed mice. Evid Based Complement Altern Med.

[40] Diraison F (2004). Over-expression of sterol-regulatory-element-binding protein-1c (SREBP1c) in rat pancreatic islets induces lipogenesis and decreases glucose-stimulated insulin release: modulation by 5-aminoimidazole-4-carboxamide ribonucleoside (AICAR). Biochem J.

[41] Fujii N (2008). Ablation of AMP-activated protein kinase α2 activity exacerbates insulin resistance induced by high-fat feeding of mice. Diabetes.

[42] Viollet B (2009). Targeting the AMPK pathway for the treatment of type 2 diabetes. Front Biosci.

[43] Salt IP, Palmer TM (2012). Exploiting the anti-inflammatory effects of AMP-activated protein kinase activation. Expert Opin Investig Drugs.

[44] Zhang Q (2016). Berberine preconditioning protects neurons against ischemia via sphingosine-1-phosphate and hypoxia-inducible factor-1α. Am J Chin Med.

[45] Chen M (2015). Berberine protects homocysteic acid-induced HT-22 cell death: involvement of Akt pathway. Metab Brain Dis.

[46] Jiang SJ (2015). Berberine inhibits hepatic gluconeogenesis via the LKB1–AMPK-TORC2 signaling pathway in streptozotocin-induced diabetic rats. World J Gastroenterol.

[47] Choi JS (2015). Anti-adipogenic effect of epiberberine is mediated by regulation of the Raf/MEK1/2/ERK1/2 and AMPKalpha/Akt pathways. Arch Pharm Res.

[48] Pires ENS (2014). Berberine was neuroprotective against an in vitro model of brain ischemia: survival and apoptosis pathways involved. Brain Res.

[49] Hsu YY, Tseng YT, Lo YC (2013). Berberine, a natural antidiabetes drug, attenuates glucose neurotoxicity and promotes Nrf2-related neurite outgrowth. Toxicol Appl Pharmacol.

[50] Datta SR (1997). Akt phosphorylation of BAD couples survival signals to the cell-intrinsic death machinery. Cell.

[51] Cardone MH (1998). Regulation of cell death protease caspase-9 by phosphorylation. Science.

[52] Saito Y (2011). Dysfunctional gastric emptying with down-regulation of muscle-specific microRNAs in *Helicobacter pylori*-infected mice. Gastroenterology.

[53] Lum JJ (2007). The transcription factor HIF-1α plays a critical role in the growth factor-dependent regulation of both aerobic and anaerobic glycolysis. Genes Dev.

[54] Morbidelli L, Donnini S, Ziche M (2003). Role of nitric oxide in the modulation of angiogenesis. Curr Pharm Des.

[55] Zhou GL (2006). Opposing roles for Akt1 and Akt2 in Rac/Pak signaling and cell migration. J Biol Chem.

[56] Liu LZ (2010). Berberine modulates insulin signaling transduction in insulin-resistant cells. Mol Cell Endocrinol.

[57] Song YC (2015). Berberine regulates melanin synthesis by activating PI3K/AKT, ERK and GSK3beta in B16F10 melanoma cells. Int J Mol Med.

[58] Chang W (2015). Berberine treatment prevents cardiac dysfunction and remodeling through activation of 5′-adenosine monophosphate-activated protein kinase in type 2 diabetic rats and in palmitate-induced hypertrophic H9c2 cells. Eur J Pharmacol.

[59] Ai F (2015). Berberine regulates proliferation, collagen synthesis and cytokine secretion of cardiac fibroblasts via AMPK-mTOR-p70S6K signaling pathway. Int J Clin Exp Pathol.

[60] Yi T (2015). Akt signaling is associated with the berberine-induced apoptosis of human gastric cancer cells. Nutr Cancer.

[61] Xiao M (2014). Berberine protects endothelial progenitor cell from damage of TNF-alpha via the PI3K/AKT/eNOS signaling pathway. Eur J Pharmacol.

[62] Ryder J, Su Y, Ni B (2004). Akt/GSK3beta serine/threonine kinases: evidence for a signalling pathway mediated by familial Alzheimer’s disease mutations. Cell Signal.

[63] Chen JH (2012). Berberine induces heme oxygenase-1 up-regulation through phosphatidylinositol 3-kinase/AKT and NF-E2-related factor-2 signaling pathway in astrocytes. Int Immunopharmacol.

[64] Bae J (2013). Berberine protects 6-hydroxydopamine-induced human dopaminergic neuronal cell death through the induction of heme oxygenase-1. Mol Cells.

[65] Fulda S (2010). Cellular stress responses: cell survival and cell death. Int J Cell Biol.

[66] Wang ZS (2010). Berberine reduces endoplasmic reticulum stress and improves insulin signal transduction in Hep G2 cells. Acta Pharmacol Sin.

[67] Li HY (2017). Berberine improves diabetic encephalopathy through SIRT1/ER stress pathway in db/db mice. Rejuvenation Res.

[68] Hao X (2012). Berberine ameliorates pro-inflammatory cytokine-induced endoplasmic reticulum stress in human intestinal epithelial cells in vitro. Inflammation.

[69] Pham TP, Kwon J, Shin J (2011). Berberine exerts anti-adipogenic activity through up-regulation of C/EBP inhibitors, CHOP and DEC2. Biochem Biophys Res Commun.

[70] Zhang W (2009). Berberine protects mesenchymal stem cells against hypoxia-induced apoptosis in vitro. Biol Pharm Bull.

[71] Brüne B (2005). The intimate relation between nitric oxide and superoxide in apoptosis and cell survival. Antioxid Redox Signal.

[72] Trachootham D (2008). Redox regulation of cell survival. Antioxid Redox Signal.

[73] Gloire G, Piette J (2009). Redox regulation of nuclear post-translational modifications during NF-kappaB activation. Antioxid Redox Signal.

[74] Liu SC (2015). Berberine attenuates CCN2-induced IL-1beta expression and prevents cartilage degradation in a rat model of osteoarthritis. Toxicol Appl Pharmacol.

[75] Zhang X (2016). Berberine activates Nrf2 nuclear translocation and inhibits apoptosis induced by high glucose in renal tubular epithelial cells through a phosphatidylinositol 3-kinase/Akt-dependent mechanism. Apoptosis.

[76] Itoh K (1997). An Nrf2 small Maf heterodimer mediates the induction of phase II detoxifying enzyme genes through antioxidant response elements. Biochem Biophys Res Commun.

[77] Hu YR (2017). Activation of Akt and JNK/Nrf2/NQO1 pathway contributes to the protective effect of coptisine against AAPH-induced oxidative stress. Biomed Pharmacother.

[78] Tan HL (2016). Rhizoma coptidis: a potential cardiovascular protective agent. Front Pharmacol.

[79] He K, Kou S, Zou Z (2016). Hypolipidemic effects of alkaloids from rhizoma coptidis in diet-induced hyperlipidemic hamsters. Immunity.

[80] Zhang X (2012). Neuroprotection of early and short-time applying berberine in the acute phase of cerebral ischemia: up-regulated pAkt, pGSK and pCREB, down-regulated NF-kappaB expression, ameliorated BBB permeability. Brain Res.

[81] Chen K (2014). Berberine reduces ischemia/reperfusion-induced myocardial apoptosis via activating AMPK and PI3K-Akt signaling in diabetic rats. Apoptosis.

[82] Youngmin K (2009). Palmatine from Coptidis rhizoma reduces ischemia-reperfusion-mediated acute myocardial injury in the rat. Food Chem Toxicol.

[83] Ashraf MI (2014). A p38MAPK/MK2 signaling pathway leading to redox stress, cell death and ischemia/reperfusion injury. Cell Commun Signal CCS.

[84] Choi JS (2014). *Coptis chinensis* alkaloids exert anti-adipogenic activity on 3T3-L1 adipocytes by downregulating C/EBP-alpha and PPAR-gamma. Fitoterapia.

[85] Jang J (2017). Berberine activates AMPK to suppress proteolytic processing, nuclear translocation and target DNA binding of SREBP-1c in 3T3-L1 adipocytes. Mol Med Rep.

[86] Yang W (2016). Jatrorrhizine hydrochloride attenuates hyperlipidemia in a high-fat diet-induced obesity mouse model. Mol Med Rep.

[87] Choi JS (2015). Protein tyrosine phosphatase 1B inhibitory activity of alkaloids from Rhizoma Coptidis and their molecular docking studies. J Ethnopharmacol.

[88] Patel MB, Mishra S (2011). Hypoglycemic activity of alkaloidal fraction of *Tinospora cordifolia*. Phytomedicine.

[89] Patel MB, Mishra S (2012). Isoquinoline alkaloids from *Tinospora cordifolia* inhibit rat lens aldose reductase. Phytother Res.

[90] Ye L (2016). Inhibition of M1 macrophage activation in adipose tissue by berberine improves insulin resistance. Life Sci.

[91] Zhao W (2017). Nandinine, a derivative of berberine, inhibits inflammation and reduces insulin resistance in adipocytes via regulation of AMP-kinase activity. Planta Med.

[92] Wang Y (2013). Attenuation of berberine on lipopolysaccharide-induced inflammatory and apoptosis responses in beta-cells via TLR4-independent JNK/NF-kappaB pathway. Pharm Biol.

[93] Zhu L, Han J, Yuan R (2018). Berberine ameliorates diabetic nephropathy by inhibiting TLR4/NF-κB pathway. Biol Res.

[94] Wang Y (2009). Berberine prevents hyperglycemia-induced endothelial injury and enhances vasodilatation via adenosine monophosphate-activated protein kinase and endothelial nitric oxide synthase. Cardiovasc Res.

[95] Xing LJ (2011). Berberine reducing insulin resistance by up-regulating IRS-2 mRNA expression in nonalcoholic fatty liver disease (NAFLD) rat liver. Eur J Pharmacol.

[96] Lou T (2011). Berberine inhibits inflammatory response and ameliorates insulin resistance in hepatocytes. Inflammation.

[97] Kong WJ (2009). Berberine reduces insulin resistance through protein kinase C-dependent up-regulation of insulin receptor expression. Metabolism.

[98] Sandeep MS, Nandini CD (2017). Influence of quercetin, naringenin and berberine on glucose transporters and insulin signalling molecules in brain of streptozotocin-induced diabetic rats. Biomed Pharmacother.

[99] Xie X (2011). Berberine ameliorates hyperglycemia in alloxan-induced diabetic C57BL/6 mice through activation of Akt signaling pathway. Endocr J.

[100] Yu Y (2010). Modulation of glucagon-like peptide-1 release by berberine: in vivo and in vitro studies. Biochem Pharmacol.

[101] Yang Z (2016). Berberine attenuates high glucose-induced fibrosis by activating the G protein-coupled bile acid receptor TGR5 and repressing the S1P2/MAPK signaling pathway in glomerular mesangial cells. Exp Cell Res.

[102] Yu Y (2015). Berberine induces GLP-1 secretion through activation of bitter taste receptor pathways. Biochem Pharmacol.

[103] Cheng Z (2006). Berberine-stimulated glucose uptake in L6 myotubes involves both AMPK and p38 MAPK. Biochim Biophys Acta.

[104] Chang W (2013). Berberine improves insulin resistance in cardiomyocytes via activation of 5′-adenosine monophosphate-activated protein kinase. Metabolism.

[105] Liu W (2009). Berberine reduces fibronectin and collagen accumulation in rat glomerular mesangial cells cultured under high glucose condition. Mol Cell Biochem.

[106] Qiu YY, Tang LQ, Wei W (2017). Berberine exerts renoprotective effects by regulating the AGEs-RAGE signaling pathway in mesangial cells during diabetic nephropathy. Mol Cell Endocrinol.

[107] Lan T (2014). Berberine attenuates high glucose-induced proliferation and extracellular matrix accumulation in mesangial cells: involvement of suppression of cell cycle progression and NF-kappaB/AP-1 pathways. Mol Cell Endocrinol.

[108] Lan T (2012). Berberine suppresses high glucose-induced TGF-beta1 and fibronectin synthesis in mesangial cells through inhibition of sphingosine kinase 1/AP-1 pathway. Eur J Pharmacol.

[109] Yerra VG (2017). Adenosine monophosphate-activated protein kinase modulation by berberine attenuates mitochondrial deficits and redox imbalance in experimental diabetic neuropathy. Neuropharmacology.

[110] Wang B (2016). Berberine improved aldo-induced podocyte injury via inhibiting oxidative stress and endoplasmic reticulum stress pathways both in vivo and in vitro. Cell Physiol Biochem.

[111] Yu SM (2016). Berberine induces dedifferentiation by actin cytoskeleton reorganization via phosphoinositide 3-kinase/Akt and p38 kinase pathways in rabbit articular chondrocytes. Exp Biol Med (Maywood).

[112] Zhao H (2014). Berberine ameliorates cartilage degeneration in interleukin-1beta-stimulated rat chondrocytes and in a rat model of osteoarthritis via Akt signalling. J Cell Mol Med.

[113] Lee YS (2010). AMP kinase acts as a negative regulator of RANKL in the differentiation of osteoclasts. Bone.

[114] Zhou K (2016). Coptisine prevented IL-beta-induced expression of inflammatory mediators in chondrocytes. Inflammation.

[115] Ishikawa S (2015). Influence of palmatine on bone metabolism in ovariectomized mice and cytokine secretion of osteoblasts. In Vivo.

[116] Hanada R (2011). RANKL/RANK—beyond bones. J Mol Med.

